# Bile acid predicts congenital portosystemic venous shunt in patients with pulmonary arterial hypertension

**DOI:** 10.1186/s40001-023-01039-0

**Published:** 2023-02-11

**Authors:** Yunyan Li, Xiaoxian Deng, Hongmei Zhou, Xuan Zheng, Gangcheng Zhang, Qingfeng Xiong

**Affiliations:** 1 Congenital Heart Disease Center, Wuhan Asia Heart Hospital, 753 Jinghan Ave, Wuhan, 430022 China; 2 Pulmonary Hypertension Center, Wuhan Asia Heart Hospital, 753 Jinghan Ave, Wuhan, 430022 China; 3 Imaging Center, Wuhan Asia Heart Hospital, 753 Jinghan Ave, Wuhan, 430022 China

**Keywords:** Congenital portosystemic venous shunt, Pulmonary arterial hypertension, Bile acid

## Abstract

The etiology of pulmonary arterial hypertension (PAH) is complex, especially the investigation of rare pathogeny is difficult. Congenital portosystemic venous shunt (CPSS) is a rare congenital anomaly in which the portal blood completely or partially bypasses the liver through a congenital portosystemic shunt, and drains directly into the inferior vena cava (IVC) (Howard and Davenport in J Pediatr Surg 32:494–497, 1997).CPSS is an uncommon cause of PAH (Christiane et al. in J Pediatr Gastroenterol Nutr 56:675–681, 2013), and often covered by other pathogenic factors. The clinical manifestations of CPSS-associated PAH are not specific, thus making it difficult to distinguish from PAH caused by other pathogenetic factors based on clinical presentations alone. This is a retrospective analysis of data from six patients with CPSS at a single center. Of these, five were diagnosed as PAH: four were also associated with other predisposing factors of pulmonary hypertension (PH). All patients had high serum bile concentration and high cardiac output. The aim of this retrospective study was to investigate the clinical recognition of PAH secondary to CPSS. The concentration of serum bile acid and cardiac output can be used as two important non-invasive indicators in clinical practice. Thus far, few studies have reported the clinical outcomes of CPSS-associated PAH specifically (Anna et al. in Hepatology 71:658–669, 2020;Franchi-Abella et al. in J Pediatr Gastroenterol Nutr 51:322–330, 2010;Uike et al. in Pediatr Pulmonol 53:505–511, 2018;). In the current study, such patients carried a poor prognosis if left untreated, or treated with pulmonary vasodilators alone.

## Case report

The data of patients who were admitted in the Pulmonary Hypertension Center of our hospital from May 2018 to July 2021 were reviewed and a total of six cases were diagnosed with CPSS:three males and three females. All of the six patients were admitted to our center because echocardiography indicated tricuspid regurgitation and the elevation of estimated pulmonary arterial systolic pressure. The main clinical manifestations of six patients included physical decline, shortness of breath, palpitation and cough, two of them had syncope history. The general conditions of the six patients were presented in Table 1. Right heart catheterization (RHC) revealed five cases of diagnosed PAH, and four of them also complicated with other possible pathogenic factors (despite one case combined with patent ductus arteriosus (PDA), PAH was not considered due to its small diameter and flow rate). All six patients had high cardiac output: cardiac index (CI) and cardiac output (CO) were increased. The results of RHC were showed in Table 2.

**Table 1:** General Information and dominant clinical features in patients

Patient	Age at CPSS diagnosis/ gender	Type of CPSS	Associated cardiac conditions	Other combined diseases	Clinical manifestation	Have PAH or not
1	19/F	CEPS: type I	PDA1.5 mm	Straight back syndrome	Cough, palpitation	YES
2	18/M	CEPS: type II	None	None	Palpitation, shortness of breath, syncope	YES
3	10/M	CEPS: type II	ASD13mm	Polymyositis (PM)/Dermatomyositis (DM)	Physical decline, shortness of breath	YES
4	20/F	CIPS	ASD16 + 9 mm	Hepatic hemangioma	Cyanosis, syncope	YES
5	4/M	CEPS: type II	None	Thrombocytopenia	Physical decline, dyspnea, chest discomfort	YES
6	31/F	CIPS	None	Mixed connective tissue disease (MCTD), Iron-deficiency anemia (IDA)	Physical decline	NO

**Table 2:** Results of echocardiography and cardiac catheterization in patients

Inspection items	Patient 1	Patient 2	Patient 3	Patient 4	Patient 5	Patient 6
LAD(cm)	2.9	3.7	2.8	2.6	2.4	3.5
LVD d/s(cm)	4.9/3.4	4.6/3.1	3.9/2.4	3.1/2	3.0/2.1	4.9/3.4
RAD(cm)	3.9	4.5	3.9	4.9	2.9	3.8
RVD(cm)	3.4	4.4	3.4	5.4	3.2	2.9
PAD(cm)	3.6	3.5	3.3	4.5	2.5	3.9
EF value (%)	56	56	60	50	58	54
TRV(max)(m/s)	3.3	4.1	5	5.9	3.3	2.3
PAP(mmHg)	50/30/37	70/35/47	77/33/48	126/77/93	50/12/29	32/12/19
aortic artery pressure(mmHg)	133/84/100	110/65/80	118/70/86	100/56/71	102/55/71	96/37/57
RAP(mmHg)	11/1/4	6/1/3	5/0/2	12/3/6	5/2/3	18/6/10
TPR(dyn·s·cm^−5^)	605	527	863	1279	114	186
PCWP(mmHg)	12	10	11	10	7	8
CO	5.9	6.87	6.1	5.59	8.7	8.65
CI	3.67	4.01	4.8	3.74	6.7	5.98
Qp/Qs	1	1.04	0.81	1.67	0.89	0.95

*LAD* Left atrial diameter, *LVD* d/s Left ventricular diameter (diastole/systole), *RAD* Right atrial diameter, *RVD* Right ventricular diameter, *PAD* Pulmonary artery diameter, *TRV* Velocity of tricuspid regurgitation, *PAP* Pulmonary artery pressure, *RAP* Right atrium pressure, *TPR* Total pulmonary resistance, *PCWP* Pulmonary capillary wedge pressure, *CO* Cardiac output, *CI* cardiac index, *Qp/Qs* Pulmonary flow/systemic flow

**Fig.1:**
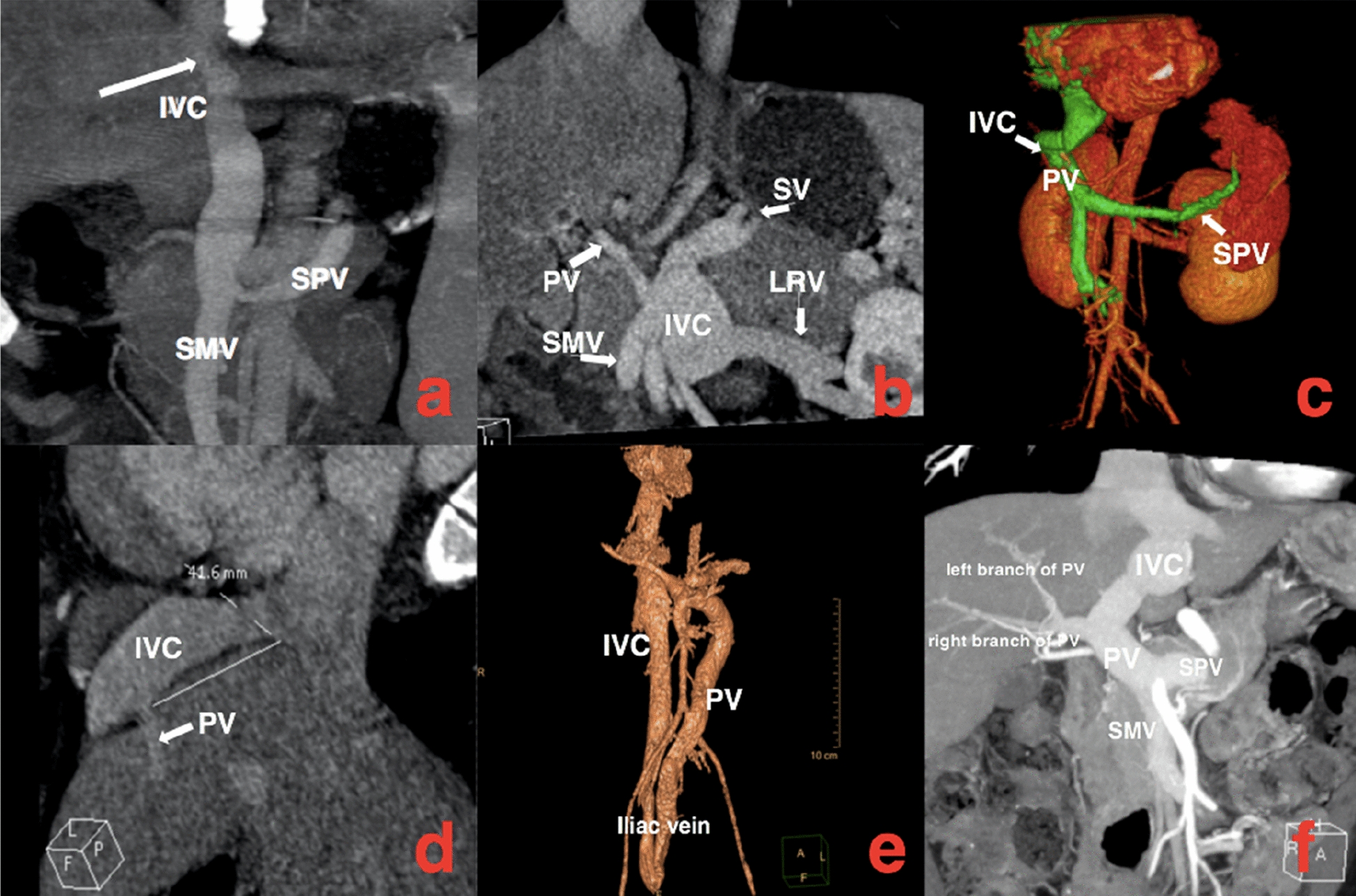
SV/SPV splenic vein, IVC inferior vena cava, PV portal vein, SMV superior mesenteric vein, LRV left renal vein. Patient 1**a** Superior mesenteric vein and splenic vein converge into portal vein, and portal vein directly flows into inferior vena cava. Patient 2**b** Superior mesenteric vein, splenic vein and left renal vein converge into portal vein, most of the portal vein flows into inferior vena cava, and another slender branch enters the liver. Patient 3**c** Most of the portal vein flows into the inferior vena cava through the left lobe of the liver, and a few into the liver. Patient 4**d** The branch of portal vein drains into inferior vena cava. Patient 5**e** Part of the portal vein flows into the inferior vena cava through the iliac vein. Patient 6**f**: Superior mesenteric vein and splenic vein converge into portal vein, the left branch of portal vein enters inferior vena cava and the right branch of portal vein enters liver

Five patients diagnosed with CPSS-associated PAH had varying degrees of liver dysfunction, including elevated alanine aminotransferase, aspartate aminotransferase and bilirubin, which were considered to be caused by liver congestion and poor function. It is noteworthy that different from the patients with PAH treated in the past, the bile acids of six patients were significantly increased. The laboratory examinations of six patients were exhibited in Table 3. Their chest radiographs indicated all had bulging pulmonary artery segments and increased pulmonary blood volume. Ultrasonic cardiogram revealed widened pulmonary artery, enlarged right atrium, tricuspid regurgitation and elevation of estimated pulmonary arterial systolic pressure as shown in Table 2. Computed tomography angiography (CTA) confirmed the fact that they had CPSS: two patients had congenital intrahepatic portosystemic shunt (CIPS) and the other patients had congenital extrahepatic portosystemic shunt (CEPS) (one type I and three type II) (Fig. 1). Five CPSS-associated PAH sufferers were followed up: Two patients underwent trans-catheter closure to block the portosystemic shunt and the pulmonary arterial pressure returned to normal. Two were managed conservatively to take pulmonary vasodilators for a long time and there were no significant changes in pulmonary artery pressure during follow-up. One patient who was recommended for surgery to close the portosystemic shunt lost follow-up.

**Table 3:** Laboratory examinations in patients

Laboratory examinations	Normal range	Patient 1	Patient 2	Patient 3	Patient 4	Patient 5	Patient 6
Serum total bile aci(μmol/L)	0–15	176.7	157.5	91.7	69.5	78.7	84
AST(IU/L)	0–50	54.2	44.8	55	22	33.9	19.4
ALT(IU/L)	0–50	35	37	34	15.9	28.1	10.9
TBIL(μmol/L)	5–21	28.5	21.9	18.3	23.9	13.3	14.3
DBIL(μmol/L)	0–3.4	8.9	6.9	5.3	4.7	3.7	2.8
NT-proBNP(pg/mL)	0–125	249	28.69	36.65	579.6	484.5	50.73

*AST* Aspartate aminotransferase, *ALT* Alanine aminotransferase, *TBIL* Total bilirubin, *DBIL* Direct bilirubin, *NT-proBNP* N-terminal brain natriuretic peptide

## Discussion

PAH refers to the clinical and pathophysiological syndrome that causes pulmonary vascular resistance and elevated pulmonary artery pressure as a result of the structural or functional changes in the pulmonary vessels caused by multiple etiologies and different pathogenesis, thereby developing into right heart failure and even death. There are many factors leading to PAH, congenital heart disease (CHD)-related PAH, heritable pulmonary arterial hypertension (HPAH) and drug‐induced pulmonary arterial hypertension (DPAH) are frequently encountered. A patient with PAH may run the risks of two or more pathogenic factors at the same time.

CPSS is an uncommon congenital portal vascular malformation which can be divided into CIPS and CEPS according to the location of abnormal vessels. CEPS is also known as Abernethy malformation (AM) as John Abernethy first reported this deformity in 1793.In 1994, Morgan and Superina [6] classified CEPS into two types in light of the absence or presence of an intrahepatic portal venous supply: Type I was congenital portal vein atresia, that was, the blood flow failed to enter the portal vein but flew into the vena cava through an abnormal channel. Based on the confluence of the superior mesenteric and splenic veins, it was sub-grouped into type Ia and type Ib. Type II was congenital portal vein dysplasia, only partial blood flow entered the portal vein and the rest flew into the vena cava through side-to-side extrahepatic communication [7–9]. Due to the diverse anatomical changes of CPSS, the clinical manifestations were also diverse and complex. The literature on CPSS combined with PAH is not common, and most of them are based on case reports [10], the pathogenesis is not completely clear. The current point has highlighted that the blood flow in the lungs increases due to the shunt of the portal venous system blood through the abnormal pathway [11]. Meanwhile, vasoactive substances (including 5-hydroxytryptamine, glucagon, histamine, estrogen, and endotoxin) produced by the visceral vascular bed enter the pulmonary circulation directly without being inactivated by the liver [12–14]. Consequently, vasodilators and vasoconstrictors cannot maintain balance in the lungs, thereby causing pulmonary vasoconstriction, remodeling and the generation of in situ thrombosis, which ultimately leads to PH [15–17]. Furthermore, some reports have revealed that heredity or genetic mutations also play a role in the occurrence and development of diseases [18].

As PAH patients with CPSS present no special clinical manifestations, it is easily to be misdiagnosed or neglected. Angiography is the gold standard for the diagnosis of CPSS. Unfortunately, it cannot be applied as a routine screening method because of its invasiveness. As the non-invasive cross-sectional imaging techniques, ultrasound, CTA or magnetic resonance imaging(MRI) can show shunts and intrahepatic portal vein branches, they can be adopted as the main means of cause screening for clinical PAH patients. Moreover, we reviewed the clinical data of six CPSS patients and found all patients had high cardiac output which was consistent with previous literature reports [19]. We also found that although some patients had varying degrees of elevated liver enzymes due to liver congestion, it is worth noting that bile acid levels were significantly elevated in all patients, whereas the patients previously diagnosed with PAH were not. There is no clear research on this by consulting the previous literature, but the consistent result was found in the data shown in an article on PAH in children with AM [20] .

The mechanism of increased bile acid in CPSS-related PAH patients is rarely reported in the literature. It may be associated with bile acid reabsorption and abnormal metabolism. Bile acid is a product metabolized from cholesterol in the liver. When blood runs through the liver, hepatocytes can uptake 60–80% of bile acids, and this is the reason why the concentration of total bile acid (TBA) in the blood of healthy individuals is very low. The increase of serum TBA concentration is the consequence of decreased liver intake and excretion (hepatocyte damage) or the result of portal system shunt. Aspartate transaminase (AST), alanine transaminase (ALT) and alkaline phosphatase (ALP) exist in cytoplasm and mitochondria, and they are enzymes reflecting liver parenchymal cell injury. In patients with CPSS-PAH, especially those with less severe elevation of pulmonary hypertension, hepatic parenchymal cells are not seriously damaged and liver enzymes may not be elevated markedly. Nevertheless, portal vein blood flows directly into the IVC completely or partially through the congenital abnormal channel, resulting in a significant increase in TBA. Therefore, we put forward this view for the first time: as a simple, non-invasive and sensitive laboratory index, bile acid is helpful in performing etiological screening of patients with PAH. When PAH patients present a remarkable increase in bile acid level, it should be alert to the existence of CPSS .

## Conclusions

Because CPSS is a rare cause of PAH, and PAH patients with CPSS are easily covered by other pathogenic factors, or be misdiagnosed as IPAH, all PAH patients need to exclude the presence or absence of CPSS. CPSS and PAH require two-way screening to avoid delays in diagnosis and treatment. As a non-invasive, fast, convenient and economical laboratory index, the level of bile acid reveals the possibility of CPSS in PAH patients. High cardiac output also serves as a reminder. Unfortunately, no unified experience or consensus has been reached on the treatment of such patients [4]. The current treatment plan rely on the type of shunt and individual circumstances. In addition to the application of standardized targeted drugs in treating PAH, a comprehensive clinical evaluation remains necessary, and even surgical management are recommended when necessary. As deeper understanding of the disease and more promising novel treatments emerge in future, the CPSS-associated PAH patients will benefit more with better therapies .

## Data Availability

Not applicable.

## Abbreviations

PAH: Pulmonary arterial hypertension
CPSS: Congenital portosystemic venous shunt
IVC: Inferior vena cava
PH: Pulmonary hypertension
RHC: Right heart catheterization
PDA: Patent ductus arteriosus
CI: Cardiac index
CO: Cardiac output
CTA: Computed tomography angiography
CIPS: Congenital intrahepatic portosystemic shunt
CEPS: Congenital extrahepatic portosystemic shunt
CHD: Congenital heart disease
HPAH: Heritable pulmonary arterial hypertension
DPAH: Drug‐induced pulmonary arterial hypertension
AM: Abernethy malformation
MRI: Magnetic resonance imaging
TBA: Total bile acid
AST: Aspartate transaminase
ALT: Alanine transaminase
ALP: Alkaline phosphatase
